# Impaired neutralizing antibodies and preserved cellular immunogenicity against SARS-CoV-2 in systemic autoimmune rheumatic diseases

**DOI:** 10.1038/s41541-022-00568-9

**Published:** 2022-11-15

**Authors:** Porntip Intapiboon, Parichat Uae-areewongsa, Jomkwan Ongarj, Ratchanon Sophonmanee, Purilap Seepathomnarong, Bunya Seeyankem, Smonrapat Surasombatpattana, Nawamin Pinpathomrat

**Affiliations:** 1 Department of Internal Medicine, Faculty of Medicine, Prince of Songkla University, Songkhla, Thailand; 2 Department of Biomedical Sciences and Biomedical Engineering, Faculty of Medicine, Prince of Songkla University, Songkhla, Thailand; 3 Department of Pathology, Faculty of Medicine, Prince of Songkla University, Songkhla, Thailand

**Keywords:** Vaccines, Translational research

## Abstract

Reports on vaccine immunogenicity in patients with systemic autoimmune rheumatic diseases (SARDs) have been inconclusive. Here, we report the immunogenicity of heterologous prime-boost with an inactivated vaccine followed by an adenoviral vector vaccine in patients with SARDs using anti-RBD antibodies, neutralizing capacity against Omicron BA.2 [plaque-reduction neutralization test (PRNT)], T cell phenotypes, and effector cytokine production at 4 weeks after vaccination. SARD patients had lower median (IQR) anti-RBD-IgG levels and neutralizing function against the Omicron BA.2 variant than the healthy group (*p* = 0.003, *p* = 0.004, respectively). T cell analysis revealed higher levels of IFN-γ- and TNF-α-secreting CD4 + T cells (*p* < 0.001, *p* = 0.0322, respectively) in SARD patients than in the healthy group. Effector cytokine production by CD8 + T cells was consistent with Th responses. These results suggest that this vaccine regimen revealed mildly impaired humoral response while preserving cellular immunogenicity and may be an alternative for individuals for whom mRNA vaccines are contraindicated.

## Introduction

Evidence of SARS-CoV-2 vaccine immunogenicity in patients with systemic autoimmune rheumatic diseases (SARDs) is sparse. In particular, neutralization of the SARS-CoV-2-Omicron BA.2 variant has not been reported, and the reported information on the cellular response has been inconclusive. Published studies have revealed that humoral immunogenicity, whether evaluated by seroconversion, anti-SARS-CoV-2 spike proteins, or neutralizing antibodies (Nabs), is reduced in patients with SARDs during immunosuppressive drugs use compared to the healthy population^1–3^. However, data on cellular immunogenicity are lacking. Prendecki et al. demonstrated preserved T-cell responses after the second dose of the ChAdOx1 nCoV-19 vaccine using an ELISpot assay^4^. Factors associated with impaired humoral immunogenicity include glucocorticoids (GC)^3,5^, rituximab^3–5^, mycophenolate mofetil (MMF)^3,5^, and abatacept^3^, while methotrexate (MTX) is associated with impaired humoral and cellular immune responses^6^.

Immunogenicity studies in SARDs have involved the homologous SARS-CoV-2 vaccine platform, while heterologous strategies have only been reported in healthy individuals. These studies demonstrated acceptable immunogenicity of ChAdOx1 nCoV-19 and BNT162b2 prime-boost vaccination regimens^7–9^. These approaches have been introduced to induce broad and sustainable immunity, particularly T cell responses, and provide maximum utilization when faced with restricted vaccine supplies or individuals for whom a homologous vaccine is contraindicated^10,11^. Recently, heterologous prime-boost vaccination with an inactivated vaccine followed with an adenoviral vector vaccine was recommended by the World Health Organization (WHO)^12^. Mahasirimongkol S et al. demonstrated higher anti-RBD-IgG and Nabs at 1-month post-vaccination with this regimen compared with vaccination with either homologous inactivated or adenoviral vector vaccines^13^. However, these findings were obtained in healthy populations, and data on the neutralization of the Omicron BA.2 variant of concern (VOC) and cellular immunogenicity are lacking.

Information on heterologous vaccination with an inactivated vaccine followed by a viral vector vaccine in patients with SARDs receiving conventional immunosuppressive drugs are lacking. Here, we evaluated the humoral immunity, particularly neutralization of the emerging Omicron BA.2 VOC, and cellular immunogenicity of heterologous vaccination with CoronaVac (Sinovac Life Sciences, Beijing, China) followed by ChAdOx1-nCoV-19 (Oxford-AstraZeneca) in patients with SARDs compared with age- and sex-match healthy group.

## Results

### Study participants

Thirty patients with SARDs and 1:1 age- and sex-matched healthy participants vaccinated with CoronaVac followed by ChAdOx1 nCoV-19 were enrolled in the study. The baseline characteristics of participants in both groups are shown in Table 1. The SARDs group comprised 86.7% females with a median (IQR) age of 41.5 (31.5–51.8) years. Systemic lupus erythematosus (SLE) was the most common SARD (50.0%), followed by rheumatoid arthritis (RA) (33.3%). Other SARDs included psoriatic arthritis (6.7%), systemic vasculitis (3.3%), systemic sclerosis (3.3%), and dermatomyositis (3.3%). Most patients were receiving GC (80.0%), with a mean (SD) prednisolone dose of 6.7 (2.8) mg per day. AZA was the most common immunosuppressive drug (43.3%), followed by MTX (40.0%) and MMF (16.7%). Five patients (16.7%) received multiple immunosuppressive drugs (two patients used MTX and AZA, three used MTX and LEF).

**Table 1:** Demographic characteristics of patients with systemic autoimmune rheumatic diseases (ARDs) and the healthy group.

Baseline characteristics	Total *N* = 60(%)	SARDs *n* = 30 (%)	Healthy group *n* = 30 (%)	*P* value
Female	52 (86.7)	26 (86.7)	26 (86.7)	1
Median age, y (IQR)	38.5 (25.5, 50)	41.5 (31.5,51.8)	35 (23,48.8)	0.181
Time to analysis, days (IQR)	32 (29,34)	32 (29,34)	32 (29,35)	0.759
SARD *n* = 30				
SLE		15 (50.0)		
RA		10 (33.3)		
Other^a^		5 (16.7)		
GC use		24 (80)		
GC dose, mean (SD)		6.7 (2.8)		
Immunosuppressive drug				
Azathioprine		13 (43.3)		
Methotrexate		12 (40)		
Mycophenolate		5 (16.7)		
Leflunomide		3 (10)		
Cyclophosphamide		1 (3.3)		
Multiple DMARDs		5 (16.7)		

*SARDs* systemic autoimmune rheumatic diseases, *DMARDs* disease-modifying antirheumatic drugs, *GC* glucocorticoids, *RA* rheumatoid arthritis, *SLE* systemic lupus erythematosus.

^a^*n* = 2 psoriatic arthritis, *n* = 1 systemic vasculitis, *n* = 1 systemic sclerosis, *n* = 1 dermatomyositis.

### Adverse events following immunization (AEFI)

AEFI is compared between patients with SARDs and the healthy group in Table 2. More than half of patients with SARDs in both groups developed at least one systemic or local reaction following vaccination with the ChAdOx1 nCoV-19 vaccine, consistent with the responses observed among healthy group. The most common systemic reaction was fever, while the most frequent local reaction was pain; however, no significant difference was observed between groups [fever: 56.7% vs. 53.3% (*p* = 1); pain: 50.0% vs. 43.3% (*p* = 1), and no patient in either group experienced a serious adverse event during the study period. There were, however, differences in AEFI between the vaccine platforms. Reactions were more common following the administration of ChAdOx1 nCoV-19 compared with the administration of CoronaVac, in terms of both local (48.3 vs. 18.3%, *p* < 0.001, respectively), and systemic (58.3 vs. 20.0%, *p* < 0.001, respectively) reactions (Table S1).

**Table 2:** Adverse events following immunization with CoronaVac followed by ChAdOx1 nCoV-19 (Oxford-AstraZeneca) in the systemic autoimmune rheumatic diseases (SARDs) group and the healthy group.

Adverse events	Total	SARDs	Healthy group	*P* value
	*N* = 60 (%)	*n* = 30 (%)	*n* = 30 (%)	
CoronaVac	16 (26.7)	10 (33.3)	6 (20)	0.381
Systemic reactions	12 (20.0)	8 (26.7)	4 (13.3)	0.333
Fever	6 (10.0)	5 (15.6)	1 (3.3)	0.197
Chill	1 (1.7)	1 (3.1)	0 (0)	1
Fatigue	3 (5.0)	2 (6.7)	1 (3.3)	1
Myalgia	2 (3.3)	2 (6.2)	0 (0)	0.492
Headache	1 (1.7)	0 (0.0)	1 (3.3)	1
Local reactions	11 (18.3)	5 (16.7)	6 (20.0)	1
Pain	9 (15.0)	4 (13.3)	5 (16.7)	1
Swelling	1 (1.7)	0 (0)	1 (3.3)	0.484
Erythema	1 (1.7)	0 (0)	1 (3.3)	0.484
Nodule	1 (1.7)	1 (3.2)	0 (0)	1
ChAdOx1 nCoV-19	43 (71.7)	21 (70.0)	22 (73.3)	1
Systemic reactions	35 (58.3)	17 (56.7)	18 (60.0)	0.1
Fever	33 (55.0)	17 (56.7)	16 (53.3)	1
Chill	7 (11.7)	2 (6.7)	5 (16.7)	0.424
Fatigue	4 (6.7)	4 (13.3)	0 (0)	0.112
Myalgia	13 (21.7)	6 (20.0)	7 (23.3)	1
Headache	3 (5.0)	2 (6.7)	1 (3.3)	1
Local reactions	29 (48.3)	15 (50)	14 (46.7)	1
Pain	28 (46.7)	15 (50)	13 (43.3)	0.796
Swelling	1 (1.7)	1 (3.3)	0 (0)	1
Erythema	0 (0)	0 (0)	0 (0)	1
Nodule	2 (3.3)	2 (6.7)	0 (0)	0.492

*SARDs* systemic autoimmune rheumatic diseases.

### SARS-CoV-2 anti-RBD antibody responses after heterologous prime-boost vaccination with an inactivated SARS-CoV-2 vaccine followed by a ChAdOx1 nCoV-19 vaccine

**Fig. 1:**
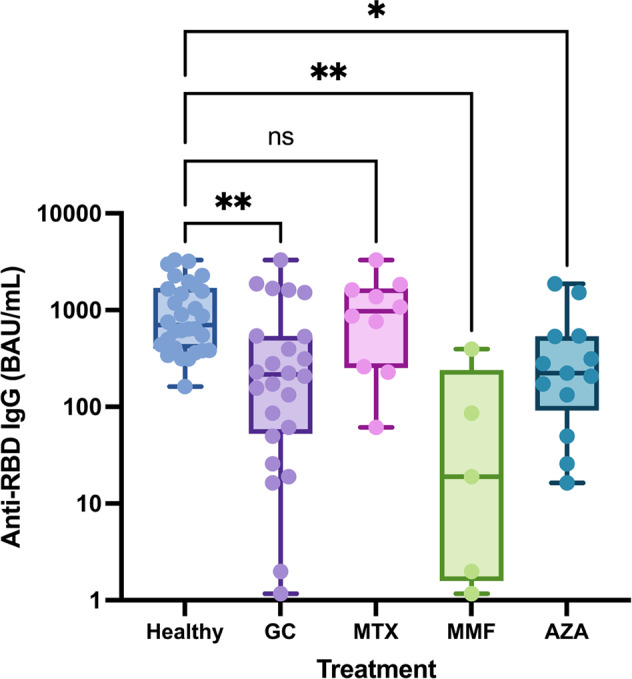
Anti-RBD IgG after CoronaVac followed by ChAdOx1 nCoV-19 (Oxford-AstraZeneca) vaccine in systemic autoimmune rheumatic diseases comparison with the healthy group. Serum samples were analyzed using chemiluminescent microparticle Immunoassay to measure anti-RBD IgG in patients with SARDs compared with the healthy group. Anti-RBD IgG levels vary by immunosuppressive drugs compared with the healthy group. Each symbol represents one participant and data are presented as the median with 95% confidence interval (CI). Statistical significance was determined using the Kruskal–Wallis test followed by Dunn’s multiple comparisons test to compare treatment groups with the healthy group. *p ≤ 0.05, **p ≤ 0.01, ns nonsignificant.

At 1-month post-vaccination, the seropositivity rate was 93.3% in the SARDs group compared with 100% in the healthy group (*p* = 0.492). SARS-CoV-2 anti-RBD antibody responses in each group are shown in Table 3. The median (IQR) anti-RBD IgG levels were significantly reduced in the sera of SARDs patients compared with those in the sera of the healthy group [270.0 BAU/mL (97.7, 1024.9) *vs* 699.5 BAU/mL (399.0, 1693.0), *p* = 0.003]. Among patients with SLE, the median (IQR) anti-RBD IgG levels were lower than those for patients with other SARDs [207.7 BAU/mL (37.7, 353.4) vs. 761 BAU/mL (200.2, 1498.4), *p* = 0.056]. SARDs patients who received GC, MMF, and AZA, demonstrated significantly impaired anti-RBD antibody responses, while methotrexate had no effect (Fig. 1). The median (IQR) anti-RBD antibody levels in GC [215.7 BAU/mL (52.6, 539.8); *p* = 0.001], MMF [19.0 BAU/mL (1.6, 240.2); *p* = 0.001], and AZA [223.7 BAU/mL (91.2, 537.6); *p* = 0.012] were lower than those in healthy group [699.5 BAU/mL (399.0, 1693.0)] (Table S2). The number of immunosuppressive drugs did not affect immunogenicity, probably due to a low immunosuppressive effect or the small group size.

**Table 3:** Immunogenicity of CoronaVac followed by ChAdOx1 nCoV-19 (Oxford-AstraZeneca) in patients with systemic autoimmune rheumatic diseases (SARDs) and the healthy group.

Immunogenicity	Total *N* = 60 (%)	SARDs *n* = 30 (%)	Healthy group *n* = 30 (%)	*P* value
Seropositivity	58	28 (93.3)	30 (100)	0.492
Anti-RBD Ab (BAU/mL); median (IQR)	547.1 (252.1,1486.6)	270 (97.7,1024.9)	699.5 (399.0,1693.0)	0.003
PRNT median (IQR)	16.5 (11,41.8)	12.0 (10,18)	29.0 (15,48.5)	0.004
Immunogenicity	SARDs *n* = 30 (%)	SLE *n* = 15 (%)	Non-SLE *n* = 15 (%)	*P* value
Seropositivity	28 (93.3)	14 (93.3)	14 (93.3)	1
Anti-RBD Ab (BAU/mL); median (IQR)	270 (97.7,1024.9)	207.7 (37.7,353.4)	761 (200.2,1498.4)	0.056

*PRNT* Plaque-reduction neutralization test, *SARDs* systemic autoimmune rheumatic diseases.

### Neutralizing capacity against the Omicron VOC after heterologous prime-boost vaccination with inactivated SARS-CoV-2 followed by ChAdOx1-nCoV-19

**Fig. 2:**
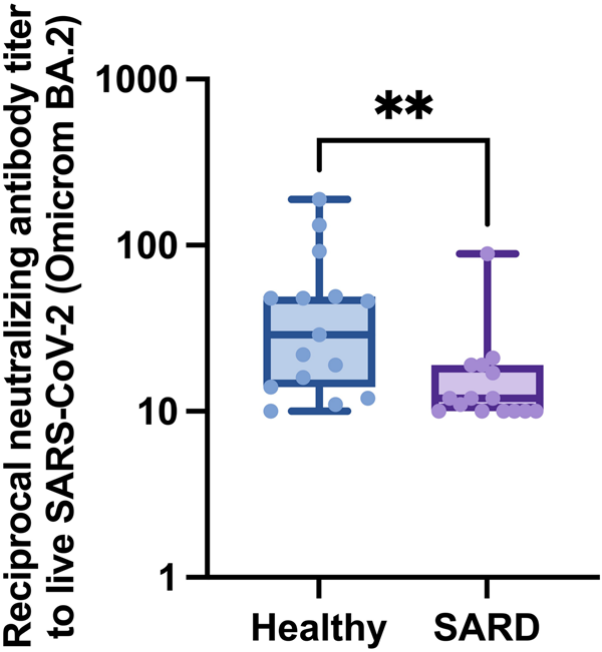
Neutralizing activity against the Omicron BA.2 variant were evaluated using PRNT. Inhibition (%) of SARS‐CoV‐2 binding to the human host receptor angiotensin-converting enzyme‐2 at 1-month after vaccination with CoronaVac (SV) followed by ChAdOx1 nCoV-19 (Oxford-AstraZeneca; AZ) in patients with SARDs and healthy group. Each symbol represents one participant, and the line denotes the median for each group (*n* = 15) with the 95% CI. Statistical significance was determined using the Mann–Whitney test. **p ≤ 0.01.

Inhibition of viral infectivity by 50% against Omicron BA.2 was evaluated by PRNT and is reported in Fig. 2. The reciprocal neutralizing titer to live SARS‐CoV‐2 Omicron BA.2 demonstrated by median (IQR) PRNT in the SARDs group was significantly lower than that in the healthy group [12.0 (10, 19) vs. 29.0 (14, 49), (*p* = 0.004)]. In addition, the PRNT results revealed markedly reduced serum antibody titers against the Omicron BA.2 VOC defined by reciprocal antibody titers <10 in 40.0% (6 of 15) of patients with SARDS compared with 6.7% (1 of 15) of healthy participants.

### T cell responses after heterologous prime-boost vaccination within activated SARS-CoV-2 followed by ChAdOx1-nCoV-19

**Fig. 3:**
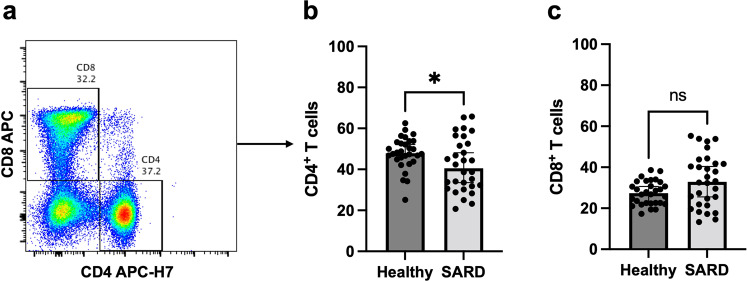
T cell responses in patients with systemic autoimmune rheumatic diseases and healthy group. Patients with SARDs and the healthy group were vaccinated with CoronaVac followed by ChAdOx1 nCoV-19 (Oxford-AstraZeneca). After 4 weeks, blood samples were obtained and processed to obtain PBMCs. Frozen PBMCs were thawed and stimulated with S1 peptide pools. The cells were stained and analyzed using flow cytometry. (**a**) Representative flow plot showing CD8 + and CD4 + T cell populations were gated. (**b**) Percentage of CD4 + T cells and (**c**) CD8 + T cells following vaccination. Each symbol represents one participant and data are presented as the median with 95% CI. Statistical significance was determined using the Mann–Whitney test between groups. *p ≤ 0.05, ns nonsignificant.

**Fig. 4:**
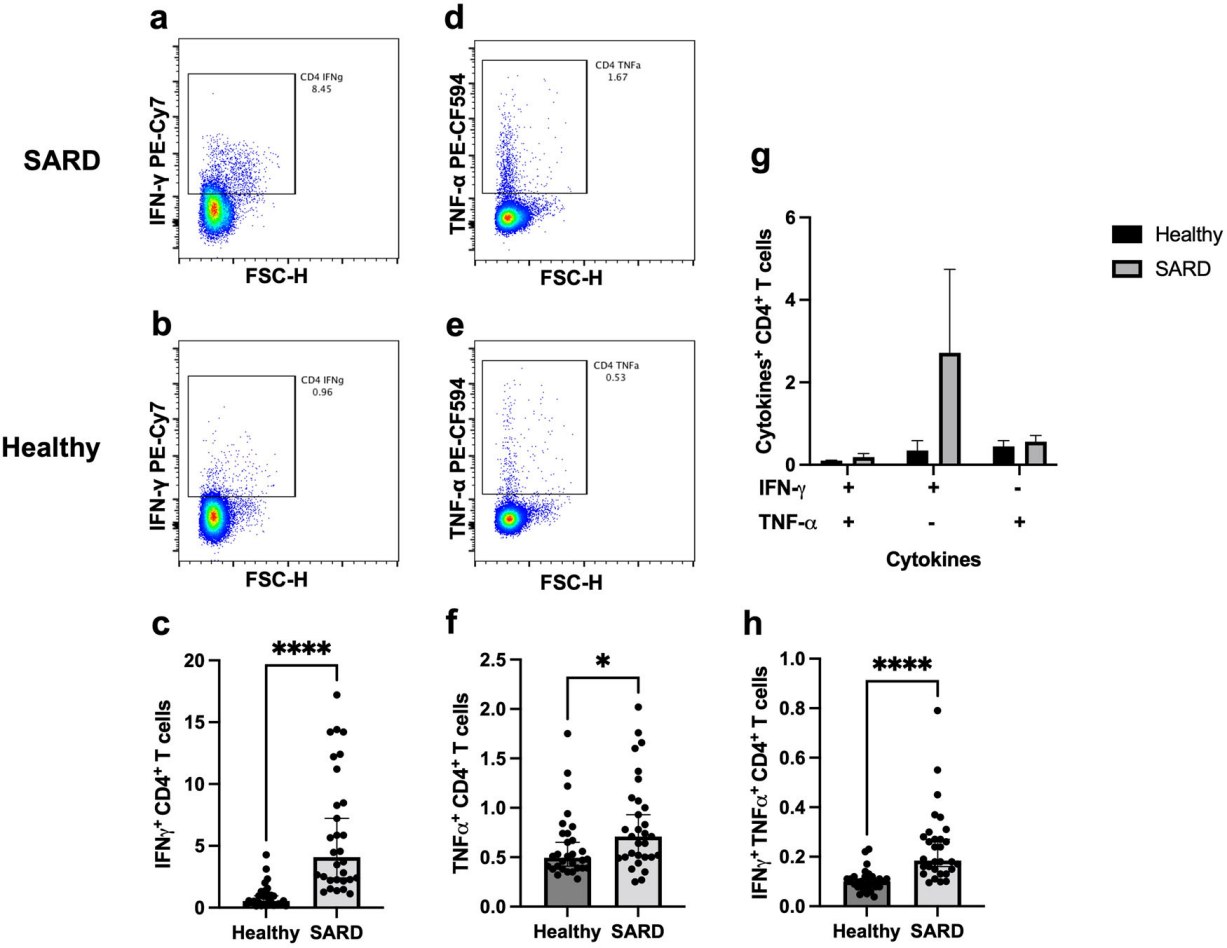
Effector cytokine-producing CD4 + T cell responses in patients with systemic autoimmune rheumatic diseases and the healthy group. Patients with SARDs and the healthy group were vaccinated with CoronaVac followed by ChAdOx1 nCoV-19 (Oxford-AstraZeneca). Four weeks after vaccination, blood samples were obtained and processed to obtain PBMCs. Frozen PBMCs were thawed and stimulated with S1 peptide pools. Blood samples were processed to obtain PBMCs. The cells were stained for surface markers and intracellular cytokines. Representative flow plots of IFN-γ producing CD4 + T cells in (**a**) patients with SARDs and (**b**) the healthy group (**c**) Percentage of IFN-γ producing CD4 + T cell responses. Representative flow plots of TNF-α producing CD4 + T cells in (**d**) patients with SARDs and (**e**) the healthy group (**f**) Percentage of TNF-α producing CD4 + T cell responses. **g** and **h** Percentages of IFN-γ and TNF-α secreting CD4 + T cells. Each symbol represents one participant and data are presented as the median with 95% CI. Statistical significance was determined using Mann–Whitney test between groups. *p ≤ 0.05, ****p ≤ 0.0001.

**Fig. 5:**
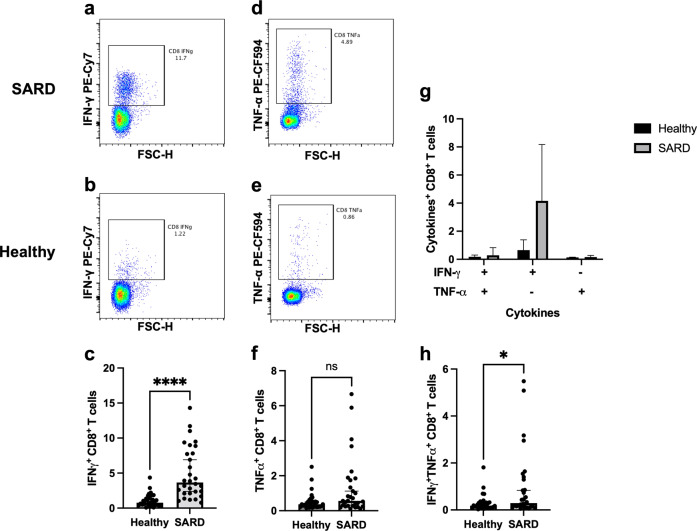
Effector cytokine-producing CD8 + T cell responses in patients with systemic autoimmune rheumatic diseases and the healthy group. Patients with SARDs and the healthy group were vaccinated with CoronaVac followed by ChAdOx1 nCoV-19 (Oxford-AstraZeneca). Four weeks post-vaccination, blood samples were obtained and processed to obtain PBMCs. Frozen PBMCs were thawed and stimulated with S1 peptide pools. The blood samples were processed to obtain PBMCs. The cells were stained for surface T cell phenotypes and intracellular effector cytokines. Representative flow plots of IFN-γ producing CD8 + T cells in (**a**) patients with SARDs and (**b**) the healthy group (**c**) Percentage of IFN-γ producing CD8 + T cell responses. Representative flow plots of TNF-α producing CD8 + T cells in (d) patients with SARDs and (**e**) the healthy group (**f**) Percentage of TNF-α producing CD8 + T cell responses. (**g** and **h**) Percentages of IFN-γ and/or TNF-α secreting CD8 + T cells. Each symbol represents one participant and data are presented as the median with 95% CI. Statistical significance was determined using Mann–Whitney test between groups. * *p* ≤ 0.05, ****p ≤ 0.0001, ns nonsignificant.

T cell responses are shown in Fig. 3. T cell population was further characterized into CD4 + and CD8 + T cells (Fig. 3a). The CD4 + T cell population was decreased in the SARDs group compared with the healthy group (Fig. 3b, *p* = 0.0426), whereas the CD8 + T cell population was comparable between the groups (Fig. 3c, *p* = 0.0817). Significantly higher levels of IFN-γ (Fig. 4a–c, *p* < 0.0001) and TNF-α (Fig. 4d–f, *p* = 0.0322) producing CD4 + T cell responses were observed in the SARDs group compared to the healthy group. Polyfunctional CD4 + T cells, which secrete both IFN-γ and TNF-α, were higher in vaccinated SARDs patients compared with the healthy individuals (Fig. 4h, *p* < 0.0001). CD8 + T cell responses followed similar trends to the T helper 1 responses (Fig. 5**)**. Heterologous prime-boost vaccination provided higher levels of cytokine-producing (IFN-γ + , TNF-α + , IFN-γ + TNF-α + ) CD8 + T cell responses in the SARDs group compared with the healthy vaccinated group (Fig. 5c, f, h, *p* < 0.0001, *p* = 0.0605, *p* = 0.0396 respectively**)**. T-cell responses in patients with SARDs using different immunosuppressive drugs and healthy group was shown in Supplementary Figure 1. The effector cytokine-producing CD4 + T cell and CD8 + T cell responses in different immunosuppressive were also shown in the Supplementary Figure 2 and Supplementary Fig. 3.

## Discussion

This study is the first to demonstrate the immunogenicity of heterologous prime-boost vaccination with an inactivated vaccine followed by the ChAdOx1 nCoV-19 viral vector vaccine in patients with SARDs receiving immunosuppressive drugs. Furthermore, we demonstrated the magnitude of humoral and cellular immunogenicity and evaluated neutralizing activity against emerging Omicron BA.2 VOC. We found an impaired humoral response and a preserved cellular immune response. Surprisingly, the levels of IFN-γ^+^ CD4^+^ T cells, IFN-γ^+^ CD8^+^ T cells, and TNFα^+^ CD4^+^ T cells were higher in the SARDs group compared to the healthy group. GC, MMF, and AZA were associated with a diminished humoral immune response. There was no difference in the AEFI between the SARDs and healthy groups.

A heterologous prime-boost or “mix and match” strategy was employed to maximize vaccine immunogenicity, and mitigate the risk for adverse reactions and vaccine shortage^14^. Using an adenovirus vector as a prime followed by other platforms as a booster improved neutralizing antibodies and Th1 T cell responses in a specific pathogen-free BALB/c mouse model^15^. Proof-of-concept heterologous vaccine studies, using the ChAdOx1-nCoV-19 vaccine boosted by BNT162b2 vaccine, revealed superior anti-RBD IgG, neutralization titer and T cell reactivity compared to homologous regimens^9,16^. Preliminary studies of heterologous prime-boost vaccination regimens with the inactivated SARS-CoV-2 vaccine followed by the ChAdOx1-nCoV-19 vaccine found that this strategy resulted in an excellent humoral response compared with homologous regimens with inactivated vaccine or adenoviral vector vaccines^13^. However, no heterologous prime-boost studies have reported outcomes for patients with SARDs. Results of our first study demonstrated the immunogenicity of a heterologous prime-boost regimen with an inactivated vaccine followed by the ChAdOx1 SARS-CoV-19 vaccine. This approach provides another option for patients with SARDs for whom homologous mRNA or adenoviral vaccines are contraindicated.

Our study demonstrated 93.3% seropositivity, which is higher than the 70.4% seropositivity obtained with a homologous inactivated vaccination regimen in 910 patients with SARDs^2^. Likewise, a 56.3% seroconversion rate was reported by Seree-aphinan et al.^17^. Data following two doses of homologous ChAdOx1 SARS-CoV-2 vaccine in patients with SARDs are lacking; however, Shenoy et al. revealed that 90.2% patients had detectable antibodies after vaccination with the ChAdOx1 SARS-CoV-19 vaccine^18^. A large case-control study of mRNA-based COVID-19 vaccines by Furer et al. reported 86% vaccine responders in the autoimmune inflammatory rheumatic diseases cohort, similar to results of Braun-Moscovici et al. and Ferri et al.^3,19^. The high seropositivity rate herein may be explained by the beneficial effect of the heterologous strategy. Nevertheless, the results may be impacted by the intensity of immunosuppressive drugs and the small number of patients. Our results revealed a seropositivity rate at least equal to homologous mRNA, or adenovirus vectors, and homologous inactivated vaccines.

The methods to evaluate the anti-SARS-CoV-2 S-protein assays are diversified, leading to difficulty comparing the results between studies. The WHO International Standard for COVID-19 serological tests aims to harmonize humoral immune response assessments using binding antibody units (BAU) as the universal reporting system. However, Infantino M et al. revealed uninterchangeable differences in commercial quantitative anti-SARS-CoV-2 S-protein assays, even with conversion to BAU/ml^20^. Thus, we could not directly compare our quantitative anti-SARS-CoV-2 S-protein assay results to those of other studies. Nevertheless, our study demonstrated a significant reduction in antibody levels compared to those of healthy groups, similar to the studies by Furer et al. and Ferri et al., which involved either the BNT162b2 or mRNA-1273 vaccine^3,19^.

Neutralizing activities were evaluated to predict vaccine efficacy; different SARS-CoV-2 vaccines utilize a wide range of Nabs. Nonetheless, reduced Nabs to the Omicron VOC is a global health problem due to its ability to escape host immunity^21^. A previous study confirmed that this variant is resistant to therapeutic antibodies and reduced neuralization capacity to double BNT162b2 vaccination^22^. No previous studies have investigated heterologous CoronaVac/ChAdOx1. Our study is the first to evaluate the neutralization of Omicron BA.2 VOC in patients with SARDs administered heterologous vaccine regimens. The results revealed significantly lower neutralizing titers in patients with SARDS compared with healthy group, and nearly half of these patients were negative in the PRNT. This finding indicated the diminished humoral vaccine immunogenicity to the Omicron VOC in patients receiving immunosuppressive drugs. These data support current recommendations for additional booster doses for patients with SARDs.

Evidence supporting vaccine-induced T cell responses is sparse and controversial. Prendecki et al. demonstrated a preserved T cell response following the completion of a second primary series of BNT162b2 mRNA or ChAdOx1-nCoV-19 vaccines in patients with SARDs. The T cell response was detected in 81.8% of patients who receiving immunosuppressive drugs, despite B-cell depletion^4^. Bitoun et al. reported a similar finding; there was no difference in CD4 T cell secreting IFN-γ and TNF levels between patients receiving immunosuppressive drugs, rituximab, and healthy group^23^. In addition, the CD8^+^-induced TNF response against spike peptides tended to be reduced in patients with a defective humoral response^23,24^. Conversely, Miyara et al. revealed a cellular response of only 57% using IFN-γ secretion levels in patients with SLE with a neutralizing antibody response after two doses of BNT162b2 vaccine^25^. Our study demonstrated that the vaccine induced a more significant polyfunctional Th1 cytokine response in both CD4 + and CD8 + T cells. These findings can be explained by the effects of ChAdOx1-nCoV-19 vaccines, which predominately induce the T cell response, and the lack of a calcineurin inhibitor. Greater Th1 cytokine secretion compared with healthy group may be explained by the baseline cellular subsets in patients with autoimmune disease^26,27^.

The use of immunosuppressive drugs contributes to impaired vaccine immunogenicity. We found that GC, MMF, and AZA were correlated with reduced anti-RBD IgG levels. The effects of GC and MMF were concordant with the previous studies^1–4^. However, MTX did not diminish immunogenicity, which contrasts to the previous findings^2,25,28,29^. This may be explained by a lower dose of MTX (10–15 mg per week) and the fact that patients temporarily withheld MTX for 1 week after each vaccine dose. Impaired humoral immunogenicity with AZA was a notable finding of our study. This effect was similar to immunogenicity reported following administration of an influenza vaccine in patients with SLE^30^. Recently, a study of the mRNA vaccine demonstrated significantly reduced antibody titers in patients with SARDs who received AZA (*p* = 0.01)^31^. Rituximab is associated with humoral impairment and strongly correlated with vaccine non-response;^3–5^ however, this study did not evaluate the impact of rituximab and instead aimed to focus on conventional immunosuppressive drugs.

Although half the patients in our cohort had SLE, we did not expect different SARD subtypes to significantly impact vaccine immunogenicity. Prior studies, which included all SARD subtypes, demonstrated that the reduction of vaccine immunogenicity depends on the intensity of immunosuppressive drugs and the vaccine platform^2,3,19^. These findings are consistent with guidelines for post-vaccination immunosuppressive drug discontinuation, in which recommendations depend on the type of immunosuppressive drug^32^. Recent studies in SLE populations revealed that MMF and GC dosage were significantly associated with impaired humoral immunogenicity after a primary series of SARS-CoV-2 vaccination^33,34^. SARS-CoV-2 vaccine immunogenicity in SLE and other SARDs were compared by Ammitzbøll et al., who reported similar seropositivity rates between 61 SLE patients and 73 rheumatoid arthritis patients after administration of an mRNA vaccine^35^. Furer et al. also demonstrated acceptable seroconversion rates in patients with SLE, RA, psoriatic arthritis and ankylosing spondylitis, while antineutrophil cytoplasmic antibody (ANCA)-associated vasculitis and idiopathic inflammatory myositis had the lowest seroconversion rates. This finding was explained by the intensity of immunosuppressive drugs, especially rituximab^3^. In summary, although the SARD subtypes in our study varied, treatment with immunosuppressive agents was found to be the major risk factor for reduced immunogenicity.

To our knowledge, this is the first prospective cohort study to compare the immunogenicity of heterologous prime-boost vaccination with inactivated vaccine followed by ChAdOx1-nCoV-19 in patients with SARDs compared with age- and sex-matched healthy controls. A strength of this study was the use of PRNT to evaluate Omicron VOC and T cell immunogenicity. Validation in larger studies analyzing the effects of each SARD subtype and immunosuppressant is required. The immunogenicity of a booster dose in this strategy is worthy of follow-up owing to the intact T-cell responses. Furthermore, these findings may support practical vaccination recommendations for patients with autoimmune rheumatic diseases who cannot be vaccinated with homologous mRNA-based vaccines.

Our study had several limitations. First, the study included a small number of patients. Second, we did not evaluate the pre-vaccination antibody status of participants; however, all patients declared no prior SARS-COV-2 infection. Third, we did not include biological or small-molecule drugs which may impact cellular immunogenicity; however, this reflects a case of real-world immunosuppressive use in populations with limited access to biological drugs. Last, because the patients did not visit the clinic simultaneously, cryopreserved cells were used for the cellular analysis. Peripheral blood mononuclear cell (PBMCs) obtained from patients treated with immunosuppressive drugs provided very limited yields. Furthermore, we did not include unstimulated controls. The responses observed could be a result of auto-reactivity as well as S1 reactivity.

Patients with SARDs who received immunosuppressive drugs, mainly GC, MMF, and AZA, had a lesser humoral vaccine response compared with healthy controls. The finding of a preserved T cell response is a highlight of this study. T cells may be essential in the prevention of severe COVID-19 disease, which is important for this valuable group. This vaccine regimen may be an option for patients with SARDs who are hesitant or have contraindications to the mRNA vaccine. However, neutralizing titer to the Omicron strain was reduced, supporting the necessity for a third booster dose.

## Methods

### Study design and participants

This prospective study was performed at the rheumatology clinic of Songklanagarind hospital, a tertiary center in Thailand, from October to December 2021. Consecutive patients with SARDs who had received ≥4 weeks of ≥1 immunosuppressive drug at a stable dose [prednisolone ≤20 mg per day, MTX ≥ 10 mg per week, leflunomide (LEF) 20 mg per day, azathioprine (AZA) ≥ 50 mg per day, and MMF ≥ 1,000 mg per day] were screened for eligibility. Patients were excluded if they had active bacterial infection, previous COVID-19 infection, were pregnant, had active malignancy, were receiving biologic drugs, intravenous immunoglobulin (IVIG), therapeutic plasma exchange (TPE), had received live virus vaccine within ≤4 weeks or inactivated vaccine within ≤2 weeks, had end-stage renal disease defined by eGFR<30 mL/min/1.73m^2^, or uncontrolled diabetes. Patients were excluded from the study if they developed active SARD, defined by the need for a dose of corticosteroid increasing to >20 mg/day, TPE, hemodialysis, or IVIG for rescue SARD therapy during the study period. All participants provided written informed consent.

Participants were administered CoronaVac followed by the ChAdOx1 nCoV-19 vaccine by intramuscular injection into the deltoid muscle, with a duration of 21–35 days between doses as indicated by the national guidelines. The patients with SARDs were advised to temporarily pause immunosuppressive drugs (AZA, MTX, MMF, and LEF), according to recommendations of the American College of Rheumatology^32^. Age- and sex-matched healthy individuals with no medical history, nor any current medication, and vaccinated under this regimen were also recruited.

### AEFIs

AEFIs were surveyed by the investigator via a questionnaire. Patients were asked about adverse events in telephone-based interviews 1 week after each vaccine dose. AEFIs were categorized into local and systemic reactions; serious reactions were classified on the basis of the medical attention required. Results were compared between groups.

### Immunogenicity of the SARS-CoV-2 vaccine

#### SARS-CoV-2 anti-RBD antibody

SARS-CoV-2 anti-RBD antibodies were evaluated 4 weeks after completing the vaccination regimen using a chemiluminescent assay against a recombinant spike (S) protein (S1/S2) by the ARCHITECT i System (Abbott, Abbott Park, IL, USA) using a chemiluminescent microparticle immunoassay (CMIA; SARS-CoV-2 IgG II Quant, Abbott Ireland, Sligo, Ireland); values exceeding 7.15 BAU/mL were considered positive.

#### PRNT

Thirty samples were randomly selected to conduct PRNT against Omicron BA.2. PRNT was performed at the Institute of Biological Products, which is a WHO-contracted laboratory at the Department of Medical Sciences. Vero cells were seeded at a density of 2 × 10^5^ cells per well and were incubated for 1 day at 37 °C in an atmosphere of 5% CO_2_. Test sera were initially diluted at ratios of 1:10, 1:40, 1:160, and 1:640. SARS-CoV-2 was diluted in the culture medium to yield 40–120 plaques/well in the control wells. Control wells, convalescent patient serum, and normal human serum were also included as assay controls. Neutralization was performed by mixing an equal volume of the diluted serum and the optimal plaque numbers of SARS CoV-2 at 37 °C in a water bath for 1 h. After removing the medium from the culture plates, the virus-serum antibody mixture (200 μL) was inoculated into the Vero monolayer and then the plates were agitated every 15 min for 1 h. Three milliliters of overlay semisolid medium (containing 1% carboxymethylcellulose [Sigma Aldrich, USA] with 1% of 10,000 units/ml penicillin–10,000 μg/ml streptomycin [Sigma Aldrich] and 10% FBS) was replaced after removing the excess virus. All plates were incubated for 7 days at 37 °C in an atmosphere of 5% CO_2_. Cells were fixed in 10% (v/v) formaldehyde and stained with 0.5% crystal violet prepared in PBS. The number of plaques formed was counted in triplicate wells, and percentage plaque reduction at 50% (PRNT50) was calculated. The PRNT50 titer of the test samples was defined as the reciprocal of the highest test serum dilution for which virus infectivity was reduced by 50% when compared to the average plaque count of the virus control; this was calculated by using a four-point linear regression method. Plaque counts for all serial serum dilutions were scored to confirm a dose response.

#### T cell responses

Flow cytometry was performed on cryopreserved PBMCs. Cells were thawed in media containing 5 U/mL benzonase and resuspended in complete RPMI medium supplemented with 10% FCS, L-glutamine, and penicillin–streptomycin (R10). Then, 1 × 10^6^ PBMCs were seeded in a 96-well plate, washed with R10, and centrifuged for 5 min at 470 × *g* and 22 °C. Each sample was stimulated with the S1 peptide pool (ProImmune), synthesized as 15-mers overlapping by ten amino acids (Supplementary Table 3). The peptide was diluted to a concentration of 2 μg/mL in R10 supplemented with anti-human CD28 and CD49d. Cells were incubated for 18 h at 37 °C with 5% CO_2_, and GolgiPlug (BD) was added after 2 h. After stimulation, the plates were centrifuged and washed with PBS. Live/Dead Aqua was diluted (1:1000 in PBS; Invitrogen) and used to stain cells for 10 min, followed by 30-minute incubation with anti-CD3, CD4, and CD8 (BD) antibodies diluted in 2% bovine serum albumin (BSA; Sigma-Aldrich) prepared in PBS (FACS buffer) (Supplementary Table 4). After surface staining, cells were fixed and permeabilized with CytoFix (BD Biosciences), in accordance with the manufacturer’s protocol. Cells were stained with anti-IFN-γ, TNF-α (BD), and diluted CytoPerm buffer (BD Biosciences) for 30 min at 4 °C, and then washed with CytoPerm buffer and resuspended in FACS buffer for analysis on a CytoflexS flow cytometer (Beckman Coulter). The acquired data was analysed using FlowJo Software (Version 10) and gated as shown in Supplementary Figure 4.

### Statistical analysis

R version 3.5.1 statistical software (R Foundation for Statistical Computing, Vienna, Austria) was used to analyze clinical data. Continuous variables were presented as the mean (SD) or median [interquartile range (IQR)], whereas categorical variables were presented as numbers and percentages. Statistical analyses of immunogenicity data were performed using GraphPad Prism 9 software (GraphPad Software Inc.). Comparisons between groups were performed using χ2, Fisher exact test or the Mann–Whitney test. The Kruskal–Wallis test, followed by Dunn’s multiple comparisons test, was performed when analyzing multiple groups. *p* < 0.05 was considered statistically significant. *p ≤ 0.05, **p ≤ 0.01, ***p ≤ 0.001, ns = non-significant.

### Reporting summary

Further information on research design is available in the Nature Research Reporting Summary linked to this article.

## Supplementary information

Supplementary

REPORTING SUMMARY

## Data Availability

Data supporting the study findings are available from the corresponding author (NP) upon request.
