# Supplementary material for: Impaired neutralizing antibodies and preserved cellular immunogenicity against SARS-CoV-2 in systemic autoimmune rheumatic diseases
Source: NPJ Vaccines. 2022 Nov 15;7:149. doi: 10.1038/s41541-022-00568-9 (PMC9666508; doi:10.1038/s41541-022-00568-9)
Supplement: Supplementary file 1 — Supplementary [file 41541_2022_568_MOESM1_ESM.pdf]

## Supplementary

**Supplementary Table 1.** Comparison of adverse events following immunization between CoronaVac and ChAdOx1 nCoV-19 (Oxford-AstraZeneca) vaccine

| | Total N=60 (%) | SV N=60 (%) | AZ N=60 (%) | Both N=60 (%) | P-value |
|---|---|---|---|---|---|
| Total AEFI | 50 (83.3) | 16 (26.7) | 43 (71.7) | 9 (15.0) | < 0.001 |
| Local AEFI | 32 (53.3) | 11(18.3) | 29 (48.3) | 7 (16.3) | < 0.001 |
| Systemic AEFI | 43 (71.6) | 12 (20.0) | 35(58.3) | 4 (6.7) | < 0.001 |

AEFI, adverse events following immunization; AZ, ChAdOx1-nCoV-19 (Oxford-AstraZeneca); SV, CoronaVac (Sinovac Life Sciences, Beijing, China)

**Supplementary Table 2.** SARS-CoV-2 anti-RBD Abs of CoronaVac followed by ChAdOx1 nCoV-19 (Oxford-AstraZeneca) vaccine according to the use of immunosuppressive drugs compared with healthy group.

| Immunosuppressive drug | SARS-CoV-2 anti-RBD Ab (BAU/mL) | P-value |
|---|---|---|
| Healthy group (n=30) | 699.5 (399.0,1693.0) | reference |
| GC (n=24) | 215.7 (52.6,539.8) | 0.001 |
| MTX (n=10) | 973.4 (252.1,1671.0) | 0.999 |
| MMF (n=5) | 19.0 (1.6,240.2) | 0.001 |
| AZA (n=13) | 223.7 (91.2,537.6) | 0.012 |

GC, glucocorticoids; MTX, methotrexate; MMF, mycophenolate mofetil; AZA, azathioprine.

**Supplementary Table 3.** Amino acid sequences of SARS-CoV-2 S1 peptides (ProImmune).

| Peptide number | Amino acid start position | Sequence |
|---|---|---|
| 1 | 1 | MFVFLVLLPLVSSQC |
| 2 | 6 | VLLPLVSSQCVNLTT |
| 3 | 11 | VSSQCVNLTTRTQLP |
| 4 | 16 | VNLTTRTQLPPAYTN |
| 5 | 21 | RTQLPPAYTNSFTRG |
| 6 | 26 | PAYTNSFTRGVYYPD |
| 7 | 31 | SFTRGVYYPDKVFRS |
| 8 | 36 | VYYPDKVFRSSVLHS |
| 9 | 41 | KVFRSSVLHSTQDLF |
| 10 | 46 | SVLHSTQDLFLPFFS |
| 11 | 51 | TQDLFLPFFSNVTWF |
| 12 | 56 | LPFFSNVTWFHAIHV |
| 13 | 61 | NVTWFHAIHVSGTNG |
| 14 | 66 | HAIHVSGTNGTKRFD |
| 15 | 71 | SGTNGTKRFDNPVLP |
| 16 | 76 | TKRFDNPVLPFNDGV |
| 17 | 81 | NPVLPFNDGVYFAST |
| 18 | 86 | FNDGVYFASTEKSNI |
| 19 | 91 | YFASTEKSNIIRGWI |
| 20 | 96 | EKSNIIRGWIFGTTL |
| 21 | 101 | IRGWIFGTTLDSKTQ |
| 22 | 106 | FGTTLDSKTQSLLIV |
| 23 | 111 | DSKTQSLLIVNNATN |
| 24 | 116 | SLLIVNNATNVVIKV |
| 25 | 121 | NNATNVVIKVCEFQF |
| 26 | 126 | VVIKVCEFQFCNDPF |
| 27 | 131 | CEFQFCNDPFLGVYY |

| Peptide number | Amino acid start position | Sequence |
|---|---|---|
| 28 | 136 | CNDPFLGVYYHKNNK |
| 29 | 141 | LGVYYHKNNKSWMES |
| 30 | 146 | HKNNKSWMESEFRVY |
| 31 | 151 | SWMESEFRVYSSANN |
| 32 | 156 | EFRVYSSANNCTFEY |
| 33 | 161 | SSANNCTFEYVSQPF |
| 34 | 166 | CTFEYVSQPFLMDLE |
| 35 | 171 | VSQPFLMDLEGKQGN |
| 36 | 176 | LMDLEGKQGNFKNLR |
| 37 | 181 | GKQGNFKNLREFVFK |
| 38 | 186 | FKNLREFVFKNIDGY |
| 39 | 191 | EFVFKNIDGYFKIYS |
| 40 | 196 | NIDGYFKIYSKHTPI |
| 41 | 201 | FKIYSKHTPINLVRD |
| 42 | 206 | KHTPINLVRDLPQGF |
| 43 | 211 | NLVRDLPQGFSALEP |
| 44 | 216 | LPQGFSALEPLVDLP |
| 45 | 221 | SALEPLVDLPIGINI |
| 46 | 226 | LVDLPIGINITRFQT |
| 47 | 231 | IGINITRFQTLLALH |
| 48 | 236 | TRFQTLLALHRSYLT |
| 49 | 241 | LLALHRSYLTPGDSS |
| 50 | 246 | RSYLTPGDSSSGWTA |
| 51 | 251 | PGDSSSGWTAGAAAY |
| 52 | 256 | SGWTAGAAAYYVGYL |
| 53 | 261 | GAAAYYVGYLQPRTF |
| 54 | 266 | YVGYLQPRTFLLKYN |
| 55 | 271 | QPRTFLLKYNENGTI |

| Peptide number | Amino acid start position | Sequence |
|---|---|---|
| 56 | 276 | LLKYNENGTITDAVD |
| 57 | 281 | ENGTITDAVDCALDP |
| 58 | 286 | TDAVDCALDPLSETK |
| 59 | 291 | CALDPLSETKCTLKS |
| 60 | 296 | LSETKCTLKSFTVEK |
| 61 | 301 | CTLKSFTVEKGIYQT |
| 62 | 306 | FTVEKGIYQTSNFRV |
| 63 | 311 | GIYQTSNFRVQPTES |
| 64 | 316 | SNFRVQPTESIVRFP |
| 65 | 321 | QPTESIVRFPNITNL |
| 66 | 326 | IVRFPNITNLCPFGE |
| 67 | 331 | NITNLCPFGEVFNAT |
| 68 | 336 | CPFGEVFNATRFASV |
| 69 | 341 | VFNATRFASVYAWNR |
| 70 | 346 | RFASVYAWNRKRISN |
| 71 | 351 | YAWNRKRISNCVADY |
| 72 | 356 | KRISNCVADYSVLYN |
| 73 | 361 | CVADYSVLYNSASFS |
| 74 | 366 | SVLYNSASFSTFKCY |
| 75 | 371 | SASFSTFKCYGVSPT |
| 76 | 376 | TFKCYGVSPTKLNDL |
| 77 | 381 | GVSPTKLNDLCFTNV |
| 78 | 386 | KLNDLCFTNVYADSF |
| 79 | 391 | CFTNVYADSFVIRGD |
| 80 | 396 | YADSFVIRGDEVRQI |
| 81 | 401 | VIRGDEVRQIAPGQT |
| 82 | 406 | EVRQIAPGQTGKIAD |
| 83 | 411 | APGQTGKIADYNYKL |

| Peptide number | Amino acid start position | Sequence |
|---|---|---|
| 84 | 416 | GKIADYNYKLPDDFT |
| 85 | 421 | YNYKLPDDFTGCVIA |
| 86 | 426 | PDDFTGCVIAWNSNN |
| 87 | 431 | GCVIAWNSNNLDSKV |
| 88 | 436 | WNSNNLDSKVGGNYN |
| 89 | 441 | LDSKVGGNYNYLYRL |
| 90 | 446 | GGNYNYLYRLFRKSN |
| 91 | 451 | YLYRLFRKSNLKPFE |
| 92 | 456 | FRKSNLKPFERDIST |
| 93 | 461 | LKPFERDISTEIYQA |
| 94 | 466 | RDISTEIYQAGSTPC |
| 95 | 471 | EIYQAGSTPCNGVEG |
| 96 | 476 | GSTPCNGVEGFNCYF |
| 97 | 481 | NGVEGFNCYFPLQSY |
| 98 | 486 | FNCYFPLQSYGFQPT |
| 99 | 491 | PLQSYGFQPTNGVGY |
| 100 | 496 | GFQPTNGVGYQPYRV |
| 101 | 501 | NGVGYQPYRVVVLSF |
| 102 | 506 | QPYRVVVLSFELLHA |
| 103 | 511 | VVLSFELLHAPATVC |
| 104 | 516 | ELLHAPATVCGPKKS |
| 105 | 521 | PATVCGPKKSTNLVK |
| 106 | 526 | GPKKSTNLVKNKCVN |
| 107 | 531 | TNLVKNKCVNFNFNG |
| 108 | 536 | NKCVNFNFNGLTGTG |
| 109 | 541 | FNFNGLTGTGVLTES |
| 110 | 546 | LTGTGVLTESNKKFL |
| 111 | 551 | VLTESNKKFLPFQQF |

| Peptide number | Amino acid start position | Sequence |
|---|---|---|
| 112 | 556 | NKKFLPFQQFGRDIA |
| 113 | 561 | PFQQFGRDIADTTDA |
| 114 | 566 | GRDIADTTDAVRDPQ |
| 115 | 571 | DTTDAVRDPQTLEIL |
| 116 | 576 | VRDPQTLEILDITPC |
| 117 | 581 | TLEILDITPCSFGGV |
| 118 | 586 | DITPCSFGGVSVITP |
| 119 | 591 | SFGGVSVITPGTNTS |
| 120 | 596 | SVITPGTNTSNQVAV |
| 121 | 601 | GTNTSNQVAVLYQDV |
| 122 | 606 | NQVAVLYQDVNCTEV |
| 123 | 611 | LYQDVNCTEVPVAIH |
| 124 | 616 | NCTEVPVAIHADQLT |
| 125 | 621 | PVAIHADQLTPTWRV |
| 126 | 626 | ADQLTPTWRVYSTGS |
| 127 | 631 | PTWRVYSTGSNVFQT |
| 128 | 636 | YSTGSNVFQTRAGCL |
| 129 | 641 | NVFQTRAGCLIGAEH |
| 130 | 646 | RAGCLIGAEHVNNSY |
| 131 | 651 | IGAEHVNNSYECDIP |
| 132 | 656 | VNNSYECDIPIGAGI |
| 133 | 661 | ECDIPIGAGICASYQ |
| 134 | 666 | IGAGICASYQTQTNS |

**Supplementary Table 4.** Fluorochrome conjugated antibodies for flow cytometry analysis.

| Antibody | Fluorochrome | Dilution | Clone | Cat number | Source |
|---|---|---|---|---|---|
| CD3 | PerCP | 1:50 | SP34-2 | 552851 | BD Horizon |
| CD4 | APC-H7 | 1:200 | SK3 | 641398 | BD |
| CD8 | APC | 1:200 | SK1 | 340584 | BD |
| L/D | Aqua | 1:1000 | - | L34957 | Invitrogen |
| IFN-gamma | PE-Cy7 | 1:100 | B27 | 557643 | BD Pharmingen |
| TNF-alpha | PE-CF594 | 1:100 | MAb11 | 562784 | BD Horizon |

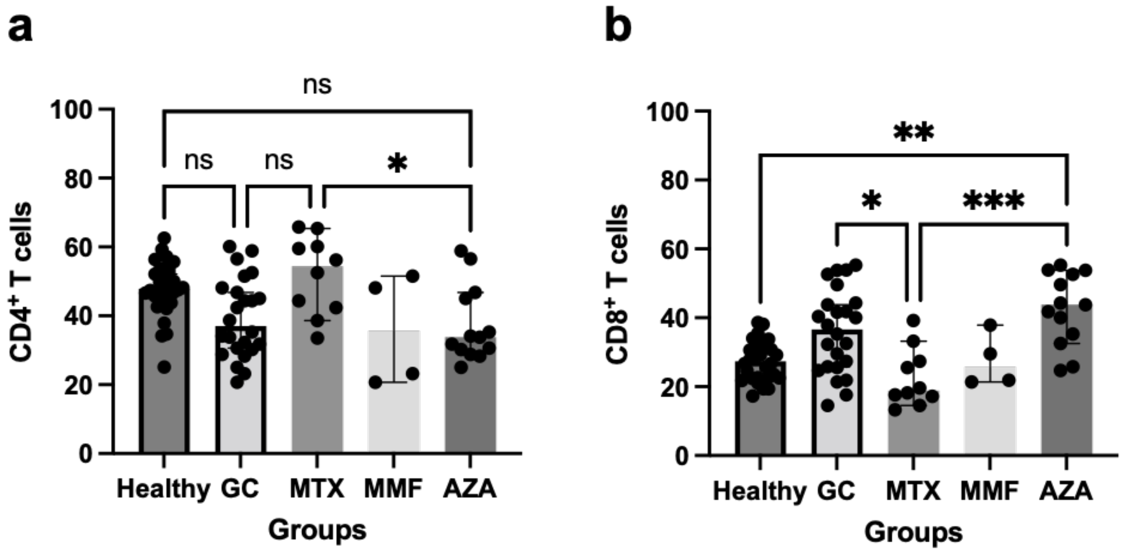


**Supplementary Figure 1.** T cell responses in patients with systemic autoimmune rheumatic diseases using different immunosuppressive drugs and healthy group. The cells were stained and analyzed using flow cytometry. T cell responses vary by immunosuppressive drugs. (a) Percentage of CD4+ T cells between each immunosuppressive drug and the healthy group.(b) Percentage of CD8+ T cells between each immunosuppressive drug and the healthy group. Each symbol represents one participant and data are presented as the median with 95% CI. Statistical significance was determined using the Mann–Whitney test between groups. *p≤0.05, **p≤0.01, ***p≤0.001, ns=non-significant.

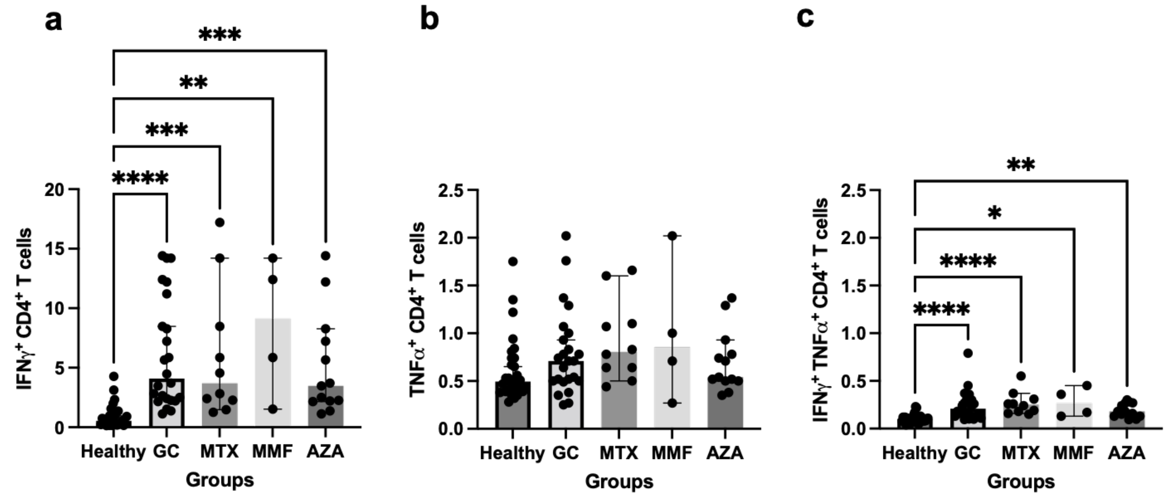


**Supplementary Figure 2.** Effector cytokine-producing CD4+ T cell responses in patients with systemic autoimmune rheumatic diseases using different immunosuppressive and healthy group. Frozen PBMCs were thawed and stimulated with S1 peptide pools. Blood samples were processed to obtain PBMCs. The cells were stained for surface markers and intracellular cytokines. Representative the flow plots of IFN-γ producing CD4+ T cell responses (a). TNF-α producing CD4+ T cells (b) and IFN-γ and/or TNF-α secreting CD4+ T cells (c) in SARDs with different immunosuppressive drugs and the healthy group. Each symbol represents one participant and data are presented as the median with 95% CI. Statistical significance was determined using Mann–Whitney test between groups. $^{*}p\leq0.05$, $^{**}p\leq0.01$, $^{***}p\leq0.001$, $^{****}p\leq0.0001$.

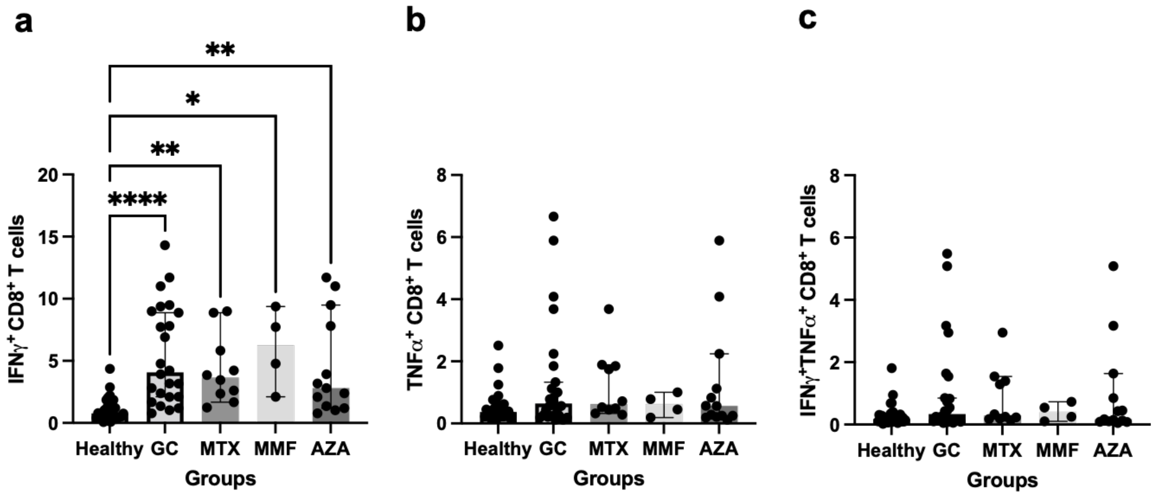


**Supplementary Figure 3.** Effector cytokine-producing CD8+ T cell responses in patients with systemic autoimmune rheumatic diseases using different immunosuppressive and healthy group. Frozen PBMCs were thawed and stimulated with S1 peptide pools. Blood samples were processed to obtain PBMCs. The cells were stained for surface markers and intracellular cytokines. Representative the flow plots of IFN-γ producing CD8+ T cell responses (a). TNF-α producing CD8+ T cells (b) and IFN-γ and/or TNF-α secreting CD8+ T cells (c) in SARDs with different immunosuppressive drugs and healthy group. Each symbol represents one participant and data are presented as the median with 95% CI. Statistical significance was determined using Mann–Whitney test between groups. * p≤0.05, ****p≤0.0001, ns=non-significant.

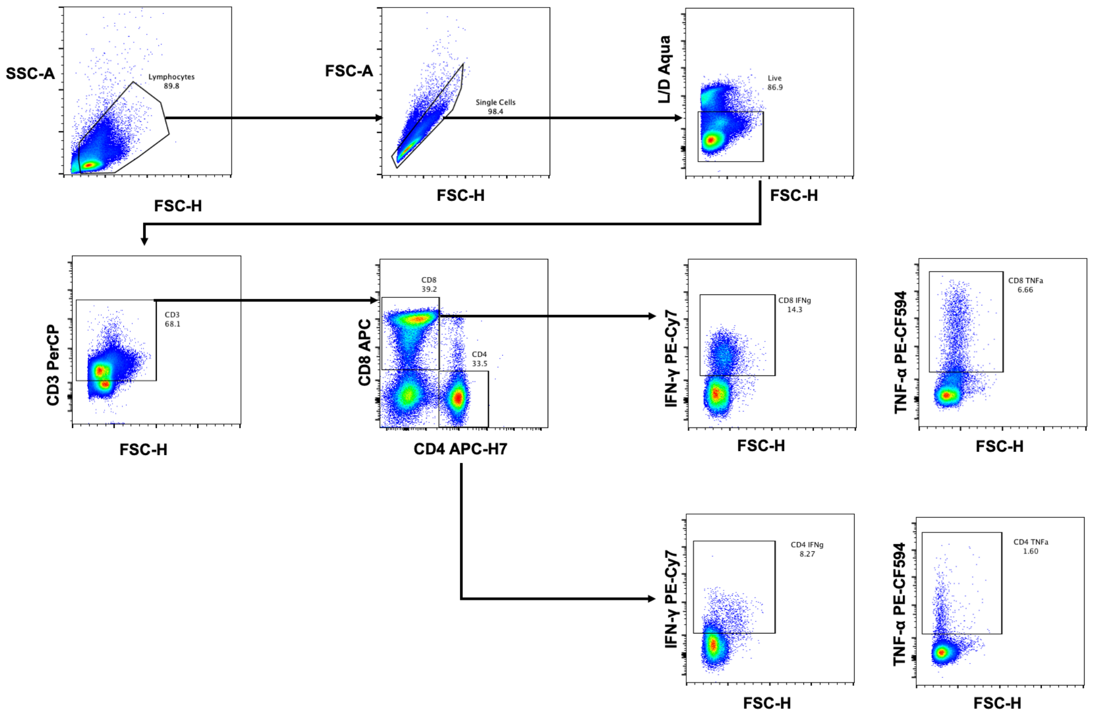


**Supplementary Figure 4.** Gating strategy for the effector cytokine producing T cells. Live CD3+ cells were gated to determine the percentages of IFN-γ and TNF-α production in CD8+ and CD4+ T cells.
