# Supplementary material for: Impaired neutralizing antibodies and preserved cellular immunogenicity against SARS-CoV-2 in systemic autoimmune rheumatic diseases
Source: NPJ Vaccines. 2022 Nov 15;7:149. doi: 10.1038/s41541-022-00568-9 (PMC9666508; doi:10.1038/s41541-022-00568-9)
Supplement: Supplementary file 2 — REPORTING SUMMARY [file 41541_2022_568_MOESM2_ESM.pdf]

nature portfolio

Corresponding author(s): NPJVACCINES-02276

Last updated by author(s): Aug 17, 2022

Reporting Summary

Nature Portfolio wishes to improve the reproducibility of the work that we publish. This form provides structure for consistency and transparency in reporting. For further information on Nature Portfolio policies, see our Editorial Policies and the Editorial Policy Checklist.

Statistics

For all statistical analyses, confirm that the following items are present in the figure legend, table legend, main text, or Methods section.

n/a	Confirmed	
☐	☒	The exact sample size (*n*) for each experimental group/condition, given as a discrete number and unit of measurement
☐	☒	A statement on whether measurements were taken from distinct samples or whether the same sample was measured repeatedly
☐	☒	The statistical test(s) used AND whether they are one- or two-sided<br>*Only common tests should be described solely by name; describe more complex techniques in the Methods section.*
☐	☒	A description of all covariates tested
☐	☒	A description of any assumptions or corrections, such as tests of normality and adjustment for multiple comparisons
☐	☒	A full description of the statistical parameters including central tendency (e.g. means) or other basic estimates (e.g. regression coefficient) AND variation (e.g. standard deviation) or associated estimates of uncertainty (e.g. confidence intervals)
☐	☒	For null hypothesis testing, the test statistic (e.g. *F*, *t*, *r*) with confidence intervals, effect sizes, degrees of freedom and *P* value noted<br>*Give P values as exact values whenever suitable.*
☒	☐	For Bayesian analysis, information on the choice of priors and Markov chain Monte Carlo settings
☒	☐	For hierarchical and complex designs, identification of the appropriate level for tests and full reporting of outcomes
☒	☐	Estimates of effect sizes (e.g. Cohen's *d*, Pearson's *r*), indicating how they were calculated

Our web collection on statistics for biologists contains articles on many of the points above.

Software and code

Policy information about availability of computer code

Data collection	Microsoft Excel version 16.56
Data analysis	R version 3.5.1 statistical software (R Foundation for Statistical Computing, Vienna, Austria), GraphPad Prism 9 software (GraphPad Software Inc.)

For manuscripts utilizing custom algorithms or software that are central to the research but not yet described in published literature, software must be made available to editors and reviewers. We strongly encourage code deposition in a community repository (e.g. GitHub). See the Nature Portfolio guidelines for submitting code & software for further information.

Data

Policy information about availability of data

All manuscripts must include a data availability statement. This statement should provide the following information, where applicable:

- Accession codes, unique identifiers, or web links for publicly available datasets
- A description of any restrictions on data availability
- For clinical datasets or third party data, please ensure that the statement adheres to our policy

Provide your data availability statement here.

Human research participants

Policy information about studies involving human research participants and Sex and Gender in Research.

Reporting on sex and gender	sex
Population characteristics	Patients with systemic autoimmune rheumatic disease,18-60 year old, who had received ≥4 weeks of ≥1 immunosuppressive drug at a stable dose [prednisolone <20 mg per day, MTX >10 mg per week, leflunomide (LEF) 20 mg per day, azathioprine (AZA) >50 mg per day, and MMF >1,000 mg per day]
Recruitment	Consecutive patients with SARDs who regular following up at rheumatology unit, of Songklanagarind hospital healthy groups were recruited by poster annoucement at vaccine center.
Ethics oversight	Human Research Ethics Committee of the Faculty of Medicine, Prince of Songkla University (REC.64– 421–14–1)

Note that full information on the approval of the study protocol must also be provided in the manuscript.

Field-specific reporting

Please select the one below that is the best fit for your research. If you are not sure, read the appropriate sections before making your selection.

☒ Life sciences ☐ Behavioural & social sciences ☐ Ecological, evolutionary & environmental sciences

For a reference copy of the document with all sections, see nature.com/documents/nr-reporting-summary-flat.pdf

Life sciences study design

All studies must disclose on these points even when the disclosure is negative.

Sample size	the sample size was determined by the data of a previous study by Geisen, U. M. et al. that evaluated the immunogenicity of anti-SARS-CoV-2 mRNA vaccines in 42 healthy controls and 26 patients with chronic inflammatory conditions undergoing immunosuppressive therapy. The IgG titers were significantly lower in patients, compared with controls (2053 BAU/mL ±1218 vs. 2685±1102 BAU/mL; 24% reduction). We calculated this using n4Studies program for testing finite population proportions. The calculated sample size (n) was 27 participants in each group with allow for a 10% dropout. The ratio between case and control is 1:1, and the final calculated sample size is 60 participants, 1:1 ratio, the total participants are 60.
Data exclusions	no
Replication	no
Randomization	this study was not a randomized control trial study. Age- and sex-matched healthy individuals with no medical history, nor any current medication, and vaccinated under this regimen were recruited as controled groups.
Blinding	this is a prospective case-control study. the blinding was not relevant to our study.

Reporting for specific materials, systems and methods

We require information from authors about some types of materials, experimental systems and methods used in many studies. Here, indicate whether each material, system or method listed is relevant to your study. If you are not sure if a list item applies to your research, read the appropriate section before selecting a response.

Materials & experimental systems

n/a	Involved in the study	
☐	☒	Antibodies
☒	☐	Eukaryotic cell lines
☒	☐	Palaeontology and archaeology
☒	☐	Animals and other organisms
☐	☒	Clinical data
☒	☐	Dual use research of concern

Methods

n/a	Involved in the study	
☒	☐	ChIP-seq
☐	☒	Flow cytometry
☒	☐	MRI-based neuroimaging

Antibodies

Antibodies used	We have provided details of all the antibodies used in this study in the online methods
Validation	We have done a flow panel optimisation before carrying out the study

Clinical data

Policy information about clinical studies

All manuscripts should comply with the ICMJE guidelines for publication of clinical research and a completed CONSORT checklist must be included with all submissions.

Clinical trial registration	this study was not registered to clinical trial.gov
Study protocol	The protocols were report in online methods
Data collection	The trial was conducted according to the principles of Good Clinical Practice by Clinical Research Center of Faculty of Medicine, Prince of Songkhla University<br>Estimated Study Start Date : October, 2021<br>Estimated Primary Completion Date : March31, 2022<br>Estimated Study Completion Date : April 30, 2022
Outcomes	Primary outcome<br>1. Compared the seropositivity rate between SARD and healthy group<br>2 .Comapared the Anti-RBD Ab levels between SARD and healthy group<br>3. Evauated the neutralization of the emerging Omicron BA.2 VOC<br>Secondary outcome<br>1. Comapare the cellular immunogenicity of heterologous vaccination with CoronaVac (Sinovac Life Sciences, Beijing, China) followed by ChAdOx1-nCoV-19 (Oxford-AstraZeneca) between SARD and healthy group

Flow Cytometry

Plots

Confirm that:

☒ The axis labels state the marker and fluorochrome used (e.g. CD4-FITC).

☒ The axis scales are clearly visible. Include numbers along axes only for bottom left plot of group (a 'group' is an analysis of identical markers).

☒ All plots are contour plots with outliers or pseudocolor plots.

☒ A numerical value for number of cells or percentage (with statistics) is provided.

Methodology

Sample preparation	Flow cytometry analysis were carried out on cryopreserved PBMCs. Cells were thawed in media containing 5 U/mL of benzonase and resuspended in complete RPMI media supplemented with 10% FCS, L-glutamine and penicillin–streptomycin (R10).
Instrument	The stained cell were analysed using CytoflexS Beckman
Software	The acquired data was analysed using FlowJo Software (Version 10)
Cell population abundance	200,000 events were recorded. Data was exported and analysed using FlowJo Software
Gating strategy	Gating strategy is provided in The supplementary information

☒ Tick this box to confirm that a figure exemplifying the gating strategy is provided in the Supplementary Information.
